# Increased urinary B2-microglobulin is associated with poor prognosis of upper tract urothelial carcinoma

**DOI:** 10.3389/fonc.2022.1008763

**Published:** 2022-10-11

**Authors:** Jang Hee Han, Seung-hwan Jeong, Si Hyun Kim, Hyeong Dong Yuk, Chang Wook Jeong, Cheol Kwak, Ja Hyeon Ku

**Affiliations:** ^1^ Department of Urology, Seoul National University Hospital, Seoul, South Korea; ^2^ Department of Urology, Seoul National University College of Medicine, Seoul, South Korea

**Keywords:** b2-microglobulin, upper tract urothelial carcinoma, disease-free survival, urinary tubular injury marker, metastasis-free survival

## Abstract

**Background:**

Kidney tubular damage markers are biomarkers of acute or chronic kidney injury. Hypothetically, upper tract urothelial cancer (UTUC), which induces obstructive uropathy or direct invasion of the renal parenchyma, may also induce increased excretion of urinary tubular damage proteins. Therefore, this study aimed to investigate the use of tubular damage biomarker as prognostic markers for UTUC.

**Methods:**

The records of 417 surgically resected patients with UTUC were obtained from the Seoul National University Prospectively Enrolled Registry for urothelial cancer-upper tract urothelial cancer (SUPER-UC-UTUC) between January 2016 and December 2020. Patients with non-urothelial cancer or without urinary tubular injury marker measurement were excluded, and finally, 296 patients were finally included. B2-microglobulin (B2-MG) was an injury marker, and a value higher than 0.3 was considered abnormally elevated, according to previous studies.

**Results:**

The mean age was 70.9 years, and the male sex was predominant (n = 211, 71.3%). The incidences of renal pelvis and ureter cancer were similar (50.7% vs. 49.3%). Most patients had high-grade diseases (n = 254, 88.8%). The high urine B2-MG group was older, had decreased renal function, and had a higher pathologic T stage than did the low group. Multivariate Cox regression analysis of disease-free survival (DFS), open surgical method (Hazard ratio (HR) 1.52, p = 0.027), large tumor size (HR 1.06, p = 0.017), tumor multifocality (HR 1.90, p = 0.038), lymphovascular invasion (HR 2.19, p < 0.001), and high urine B2-MG (HR 1.57, p = 0.021) were significantly associated with shortened metastasis-free survival (MFS). Kaplan–Meier curve analysis revealed short DFS (median survival 15.5 months vs. unattained, log-rank p = 0.001) and MFS (unattained median survival in both groups, log-rank p = 0.003) for the high urine B2-MG group compared to the low urine B2-MG group.

**Conclusion:**

Patients with UTUC presenting with increased pre-operative urine B2-MG levels were associated with disease recurrence and metastasis. This biomarker may aid in performing pre-operative risk stratification and in assessing the individual prognosis of patients with UTUC.

## Introduction

Urothelial cell carcinoma, which comprises upper tract urothelial carcinoma (UTUC) and bladder cancer, is the fourth most common tumor with a steady increase in incidence (1, 2). Due to the histologic similarities between both cancers (UTUC and bladder cancer), clinical decision-making for UTUC is usually based on that for bladder cancer (3). However, UTUC has different clinical manifestations compared to bladder cancer in that it has a much higher rate of muscle invasion at diagnosis (60% of UTUC compared to 15–25% of bladder cancer) (4). This leads to a higher incidence of advanced disease and a poor 5-year survival rate, which is < 50% for patients with T2–T3 stage and < 10% for those with T4 stage (5). Several pathologic factors, such as tumor stage, tumor grade, and lymphovascular invasion, are associated with patients’ prognoses; however, current classifications cannot perfectly stratify risk levels compared to the well-validated risk stratification tools for bladder cancer (6).

The surrounding environment of UTUC differs from that of bladder cancer: The intraluminal space is much smaller, which offers a higher chance of ipsilateral urinary tract obstruction, leading to ipsilateral hydronephrosis. Moreover, tumors in the renal pelvis have a unique interface with the renal parenchyma, which may directly invade it as the tumor grows. Interestingly, both direct tumor infiltration and indirect induction of kidney damage by urinary compression or obstruction cause kidney injury (7), which may be associated with increased excretion of urinary tubular damage markers.

Thus, this study hypothesized that an increased urinary B2-microglobulin (B2-MG) (available tubular injury marker in this study) might represent the tumor burden or aggressive behavior of UTUC. Furthermore, we assessed the prognostic value of pre-operative urinary B2-MG for disease-free survival (DFS) and metastasis-free survival (MFS).

## Materials and methods

### Ethics approval and informed consent

The Institutional Review Board (IRB) of the Seoul National University Hospital approved this study (IRB no. 2207-089-1340). The requirement for informed consent was waived owing to the retrospective study design. The study was performed in accordance with applicable laws and regulations, good clinical practice, and ethical principles, in accordance with the Declaration of Helsinki.

### Patient population

We reviewed patients enrolled in the Seoul National University Prospectively Enrolled Registry for Urothelial Cancer-Upper Tract Urothelial Cancer (SUPER-UC-UTUC) who underwent surgical resection between January 2016 and December 2020 at the Seoul National University Hospital. During this period, 413 patients were included in the database. Patients diagnosed with non-urothelial cancer and those without pre-operative urine B2-MG measurements were excluded. Eventually, 296 patients were included in the analysis.

### Collected parameters and definition of increased urine B2-MG

Patients’ demographic and clinicopathological data, including sex, age at surgery, comorbidities, pre-operative imaging findings, pre-operative laboratory findings (serum creatinine, estimated glomerular filtration rate [eGFR], urine B2-MG, pathological histology, grade, stage, and lymphovascular invasion), and disease statuses (DFS and MFS) were collected. Furthermore, increased urinary B2-MG levels (>0.3) were defined based on the previous definition by Bethea et al. (8).

### Statistical analysis

The clinical and pathological characteristics of patients with low urine B2-MG (≤ 0.3) and high urine B2-MG (> 0.3) were compared using the independent Student’s *t*-test and chi-square test. Additionally, univariate and multivariate Cox regression analyses assessed the independent influence of the possible risk factors on DFS and MFS. All statistical analyses were performed using SPSS version 25 software (SPSS, version 25.0.0.2, IBM Corp., Armonk, NY, USA). Statistical significance was set at a p-value < 0.05, and all the tests were two-sided.

## Results

This study enrolled 296 patients with a mean age of 70.9 ± 10.0 years and a median follow-up of 19.7 months. The male sex was predominant (n = 211, 71.3%), and the mean body mass index (BMI) was 24.7 ± 3.3 kg/m^2^. Almost an equal number of patients with renal pelvis cancer (n = 150, 50.7%) and urothelial ureter cancer (n = 146, 49.3) were included. Furthermore, based on pre-operative laboratory results, the mean creatinine level was 1.21 ± 1.05 mg/dL, the mean eGFR was 64.7 ± 20.8 mL/min/1.73 m^2^, and the median urine B2-MG was 0.27 ug/mL (**Table 1**).

**Table 1:** Baseline characteristics.

Total (n)	296
Age (years) (mean ± SD)	70.9 ± 10.0
Sex (Male) (n, %)	211 (71.3)
BMI (kg/m^2^) (mean ± SD)	24.7 ± 3.3
HTN (n, %)	175 (59.1)
DM (n, %)	85 (28.7)
*Smoking status*	
Non-smoker	144 (48.6)
Smoker	152 (51.4)
Laterality (Right)	130 (43.9)
Tumor size (cm)	3.7 ± 2.7
*Tumor location (n, %)*	
Renal pelvis	150 (50.7)
Ureter	146 (49.3)
Multiplicity (n, %)	21 (7.1)
*Pre-operative laboratory tests*	
Creatinine (mg/dL)	1.21 ± 1.05
eGFR (mL/min/1.73 m^2^)	64.7 ± 20.8
Urine B2-MG	0.27 [0.19, 0.74]
Median follow-up period (months)	19.7 [10.2, 34.2]

BMI, body mass index; eGFR, estimated glomerular filtration rate; B2-MG: beta-2 microglobulin; HTN, hypertension; BMI, body mass index; DM, Diabetes mellitus.

**Figure 1:**
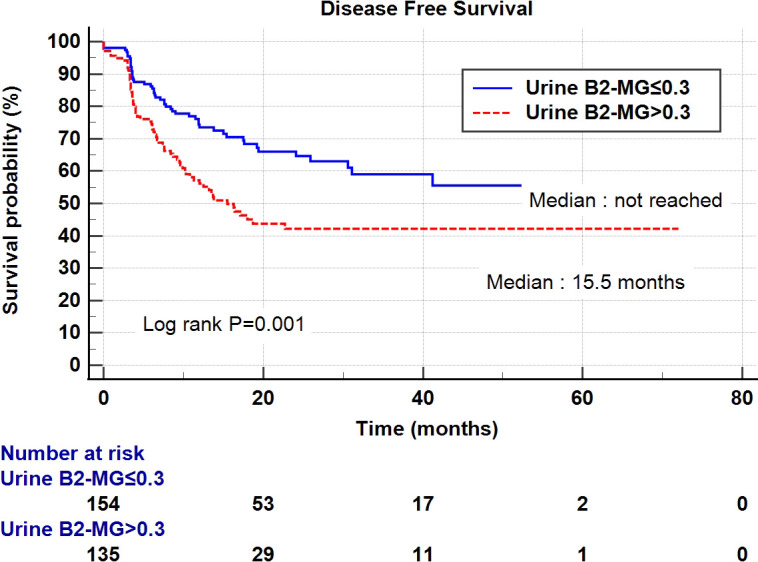
Kaplan-Meier curve of the effect of low urine B2-MG (≤ 0.3) (blue) and high urine B2-MG (> 0.3) (dotted red) groups on disease-free survival.

**Figure 2:**
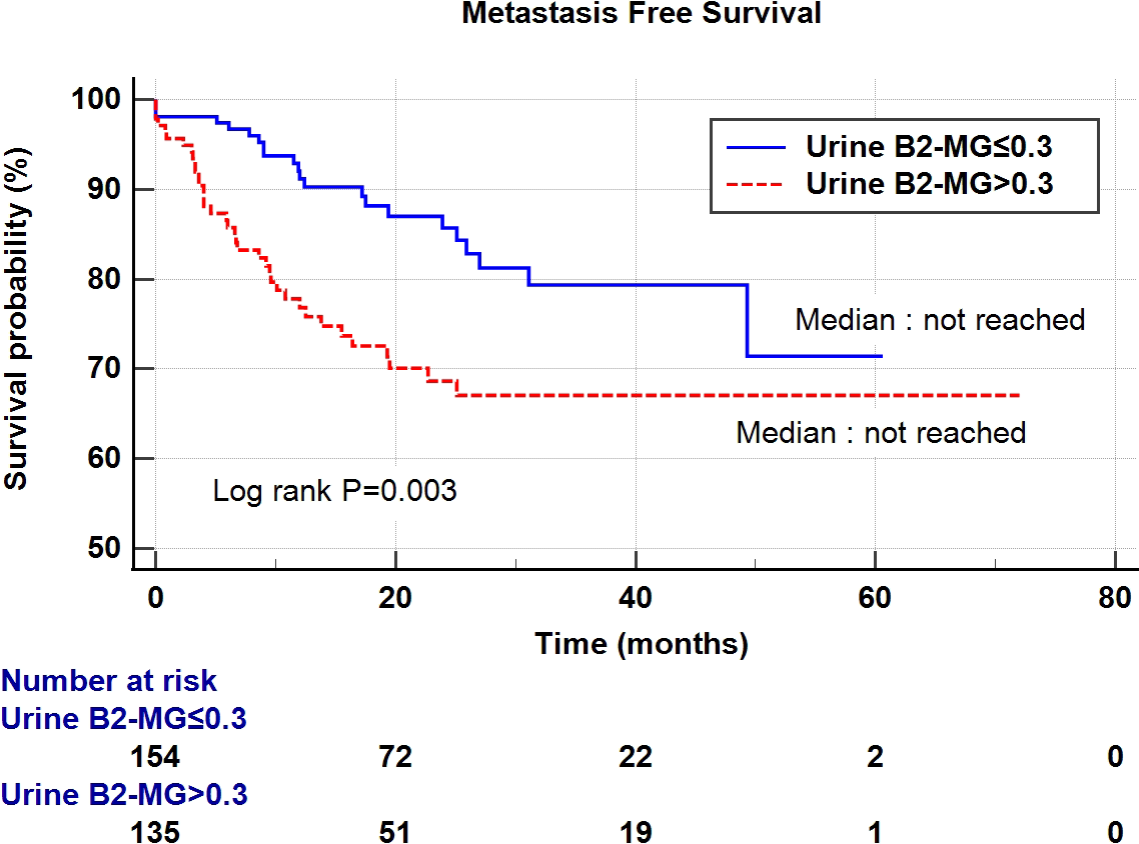
Kaplan-Meier curve of the effect of low urine B2-MG (≤ 0.3) (blue) and high urine B2-MG (> 0.3) (dotted red) groups on metastasis-free survival.

Regarding the perioperative findings, the high-grade disease was predominant (n = 254, 88.8%). The number of patients with high pathological T stage (T2–T4) (n = 155, 52.4%) and low T stage (Ta–T1) (n = 141, 47.6%) was similar. Lymphovascular invasion was observed in 45 patients (15.2%), whereas carcinoma *in situ* (CIS) was observed in 83 patients (28.0%) (**Table 2**). The patients were divided into two subgroups according to their urine B2-MG levels. Low urine B2-MG levels (≤ 0.3) were observed in 157 patients, while high urine B2-MG levels (> 0.3) were detected in 139. Patients in the high urine B2-MG group were significantly older (p = 0.004), at risk for hypertension (p = 0.037), and had a decreased eGFR (p < 0.001). Regarding oncological characteristics, tumor size was larger (4.23 ± 3.34 cm vs. 3.23 ± 1.96 cm in the high and low B2-MG groups, respectively, p = 0.002), pathologic T stage was higher (T2–T4; 61.2% vs. 44.6% in the high and low B2-MG groups, respectively, p=0.004), and accompanied perineural invasion more often (11.1% vs. 3.2% in the high and low B2-MG groups, respectively, p=0.009) (**Table 3**). Univariate and multivariate Cox regression analyses evaluated the risk factors for disease recurrence and metastasis. The results revealed that the open surgical method (Hazard ratio (HR) 1.52, 95% confidence interval [CI] 1.05–2.20, p = 0.027), large tumor size (HR 1.06, CI 1.01–1.11, p = 0.017), tumor multifocality (HR 1.90, CI 1.04–3.48, p = 0.038), presence of lymphovascular invasion (HR 2.19, CI 1.391–3.45, p < 0.001), and high urine B2-MG (HR 1.57, CI 1.075–2.31, p = 0.021) were significantly associated with disease recurrence (**Table 4**). Regarding metastatic events, a higher T stage (≥ T2) (HR 3.41, CI 1.49–7.81, p = 0.004), lymphovascular invasion (HR 4.21, CI 2.42–7.32, p < 0.001), and high urine B2-MG (HR 1.78, CI 1.03–3.07, p = 0.039) were associated with metastasis (**Table 5**). On Kaplan–Meier curve analysis, high urine B2-MG presented significantly shorter DFS (median survival 15.5 months vs. unattained, p = 0.001) (**Figure 1**) and MFS (median survival unattained for both groups, p = 0.003) (**Figure 2**).

**Table 2:** Perioperative characteristics.

Total (n)	296
*Surgical method (n, %)*	
Open	122 (41.2)
Minimally invasive surgery	173 (58.8)
*Surgical type*	
Segmental ureterectomy	12 (4.1)
Nephroureterectomy with bladder cuff resection	284 (95.9)
Operative time (minutes)	146.6 ± 60.3
Estimated blood loss (mL) (IQR)	150 [100, 300]
Venous invasion (n, %)	23 (7.8)
Lymphovascular invasion (n, %)	45 (15.2)
Perineural invasion (n, %)	20 (6.8)
Carcinoma *in situ* (n, %)	83 (28.0)
*Grade (n, %)*	
High grade	254 (88.8)
Low grade	32 (10.8)
*Pathologic T stage (n, %)*	
Ta-T1	141 (47.6)
T2-4	155 (52.4)
*Pathologic N stage (n, %)*	
N0	40 (13.5)
N1-2	15 (5.1)
Nx	241 (81.4)

**Table 3:** Comparison of low and high urine B2-MG groups.

	Low urine B2-MG (≤ 0.3) (n =157)	High urine B2-MG (> 0.3) (n = 139)	p-value
Age (years)	69.3 ± 10.0	72.6 ± 9.6	0.004
Sex (Male) (n, %)	114 (72.6)	97 (69.8)	0.592
BMI (kg/m^2^) (mean ± SD)	24.8 ± 3.6	24.6 ± 3.0	0.528
HTN (n, %)	84 (53.5)	91 (65.5)	0.037
DM (n, %)	51 (32.5)	34 (24.5)	0.128
Creatinine (mg/dL)	1.08 ± 0.67	1.35 ± 0.82	0.003
eGFR (mL/min/1.73 m^2^)	71.0 ± 19.9	57.6 ± 19.6	<0.001
Hydronephrosis (n, %)	72 (45.9)	78 (56.1)	0.078
Laterality (Right)	66 (42.0)	64 (46.0)	0.488
Tumor size (cm)	3.23 ± 1.96	4.23 ± 3.34	0.002
*Tumor grade (n, %)*			0.479
Low grade	19 (12.4)	13 (9.8)	
High grade	134 (87.6)	120 (90.2)	
*Pathologic T stage (n, %)*			0.004
Ta-T1	87 (55.4)	54 (38.8)	
T2-4	70 (44.6)	85 (61.2)	
*Pathologic N stage (n, %)*			0.560
N0	16 (10.2)	24 (17.3)	
N1-2	8 (5.0)	7 (5.0)	
Multiplicity (n, %)	8 (5.1)	13 (9.4)	0.209
Perineural invasion (n, %)	5 (3.2)	15 (11.1)	0.009
Carcinoma *in situ* (n, %)	43 (27.9)	40 (29.6)	0.749
Lymphovascular invasion (n, %)	21 (13.6)	24 (17.8)	0.333

BMI, body mass index; eGFR, estimated glomerular filtration rate; B2-MG: beta 2 microglobulin; HTN, hypertension; BMI, body mass index; DM, Diabetes mellitus.

**Table 4:** Univariate and Multivariate Cox regression analysis of disease-free survival.

	Univariate Analysis			Multivariate Analysis		
Variables	HR	95% CI	p-value	HR	95% CI	p-value
*Disease-free Survival*						
Age (years)	0.79	0.98–1.02	0.997			
Sex (Male)	1.22	0.81–1.84	0.334			
Open Surgical method	1.56	1.09–2.25	0.016	1.52	1.05–2.20	0.027
Tumor size (cm)	1.07	1.02–1.11	0.003	1.06	1.01–1.11	0.017
Multifocality	2.26	1.24–4.12	0.008	1.90	1.04–3.48	0.038
High grade	1.48	0.78–2.84	0.234			
T stage ≥ T2	1.66	1.14–2.42	0.008	1.24	0.83–1.87	0.297
Concomitant CIS	1.46	0.99–2.13	0.055			
Lymphovascular invasion	2.21	1.45–3.67	<0.001	2.19	1.39–3.45	0.001
eGFR < 60 (mL/min/1.73 m^2^)	1.38	0.96–1.99	0.079			
Urine B2-MG (> 0.3 ug/mL)	1.74	1.20–2.53	0.004	1.57	1.07–2.31	0.021

CI, confidence interval; CIS, carcinoma in situ; eGFR, estimated glomerular filtration rate.

**Table 5:** Univariate and Multivariate Cox regression analysis of metastasis-free survival.

	Univariate Analysis			Multivariate Analysis		
Variables	HR	95% CI	p-value	HR	95% CI	p-value
*Disease-free Survival*						
Age (years)	0.99	0.97–1.03	0.954			
Sex (Male)	0.94	0.54–1.62	0.818			
Open Surgical method	1.58	0.94–2.65	0.085			
Tumor size (cm)	1.06	0.99–1.13	0.099			
Multifocality	1.76	0.75–4.10	0.192			
High grade	8.32	1.15–60.15	0.036	2.10	0.26–17.09	0.489
T stage ≥ T2	6.16	3.02–12.55	<0.001	3.41	1.49–7.81	0.004
Concomitant CIS	1.55	0.914–2.63	0.104			
Lymphovascular invasion	6.27	3.72–10.56	<0.001	4.21	2.42–7.32	<0.001
eGFR < 60 (mL/min/1.73 m^2^)	2.35	1.39–3.97	0.001	1.61	0.94–2.77	0.086
Urine B2-MG (> 0.3 ug/mL)	2.18	1.29–3.70	0.004	1.78	1.03–3.07	0.039

CI, confidence interval; CIS, carcinoma in situ; eGFR, estimated glomerular filtration rate.

## Discussion

Renal tubular damage markers are widely used in clinical practice as noninvasive tools for assessing acute or chronic kidney injury induced by various causes (9–11). Its role in oncology has not been highlighted; however, it has been used in pediatric urology for a long time to evaluate the impact of congenital diseases, such as vesicoureteral reflux or ureteropelvic junction obstruction, on kidney parenchymal damage (12, 13). This is essential because it provides clinicians with clues on determining whether the patient needs surgical correction and planning the treatment strategy before operation. Tubular damage markers have recently gained attention for several cancers. Zhang et al. discovered that kidney injury molecule-1 (KIM-1) might be a surrogate biomarker for kidney cancer (14). Lucarelli et al. demonstrated that patients with kidney cancer having higher B2-MG levels exhibited poor progression-free survival (15). Moreover, Aviles et al. demonstrated that an increased B2-MG pretreatment level is one of the most useful prognostic factor in patients with multiple myeloma (16). Shang et al. revealed that B2-MG is an independent prognostic factor in T-cell lymphoma (17). Therefore, we hypothesized that UTUC might directly or indirectly affect the ipsilateral kidney, inducing the excretion of the tubular damage marker, B2-MG. There is currently no appropriate pre-operative prognostic factor; hence, we aimed to discover novel prognostic factors based on the pathophysiology of UTUC, which may not be detected through conventional radiographic imaging studies.

B2-MG is a single-chain low-molecular-weight polypeptide, and due to its small size, it is freely filtered through the kidney glomeruli, and mostly reabsorbed by the renal proximal tubular cells (18). Thus, under normal conditions, only trace amounts of β2-microglobulin remain in the urine and are excreted (19). However, in pathologic conditions, such as UTUC in this study, there is a higher chance of reabsorption disturbance due to increased tubular damage. And its risk is thought to increase as tumor progresses or possess invasive features. As the basis of this theory, two types of tumor cell proliferation may help to explain; the less invasive type is the type of tumor displacing the normal structure, causing mild compression damage to the surrounding healthy tissues (normal kidney tubules in this study); the other type is the invasive type with the tumor invading or infiltrating the surrounding healthy tissues (20), leading to architectural disruption, which results in significant tubular damage. Consistent with this theory, our results revealed that the high urine B2-MG group presented a higher incidence of pathological invasive features, such as perineural invasion and higher T stage (61.2% vs 44.6%, p=0.004). In the same context that hydronephrosis induces increased urinary B2-MG (21), a higher tumor burden or mass effect (large size) (4.23 ± 3.34 vs 3.23 ± 1.96, p=0.002) resulting in ipsilateral urinary stasis may also induce kidney tubular damage and increase B2-MG excretion.

As urine B2-MG is affected by multiple clinical factors, the independent effect of increased urine B2-MG on UTUC prognosis requires further investigation to exclude confounding factors. From the previous studies, older age (22), accompanying hypertension (23, 24), and reduced renal function (25) is known to be related to the increased urine B2-MG levels, which was consistently reproduced in our prospective cohort. Furthermore, hydronephrosis induces kidney damage, thus increasing urine B2-MG excretion, as previously mentioned (21). Bozzini et al. demonstrated poor prognosis in patients with ipsilateral hydronephrosis induced by UTUC (26). Indeed, patients with ipsilateral hydronephrosis had significantly higher B2-MG level compared to others (2.39 ± 8.97 vs 0.81 ± 2.12, p=0.038) in our study. However, Cox regression analysis revealed that increased urine B2-MG was associated with disease prognosis independently of the influence of the above variables. Above findings indicate that urine B2-MG has an independent impact on disease recurrence and metastasis associated with intrinsic tumor factors.

Our prospective cohort study had several unique characteristics. Male patients were predominant, and the mean age of the patients was comparable to the known peak age of UTUC (27). The relative proportion of UTUC in the renal pelvis and ureter was also consistent with a previous report (28). Furthermore, approximately 90% of the patients harbored high-grade diseases, and thus, the assessment of the prognostic effect of disease grade did not have high statistical power. Previous studies reported that patients with ureteral cancer presented worse outcomes than those of patients with renal pelvic cancer (29); however, our cohort did not reveal a significant difference (p = 0.811 for DFS, p = 0.879 for MFS). We believe that this difference can be attributed to two reasons. First, incidence data from previous studies were collected from a retrospective database and may not reflect real-time data. Second, this may be the result of the increasing trend of high-grade UTUC, as evidenced by a previous study by Thomas et al. (30). Regarding the surgical method, we performed nephroureterectomy using an open or minimally invasive approach. There is conflicting evidence regarding the impact of the surgical method on prognosis; therefore, we further analyzed its impact and demonstrated that in our cohort the open method was significantly associated with worse DFS, but not MFS. Furthermore, as updated recent multicenter study by ROBUUST group (31), robotic and laparoscopic surgery group showed comparable oncological outcomes. Recent systemic review and meta-analysis demonstrate that advanced tumor stage and lymphovascular invasion as the most prognostic factor for overall survival (32). Our cohort also showed consistent result, and we additionally found that B2-MG could be a novel prognostic factor having the comparable prognostic power with above two factors. Collectively, our prospective cohort reflected real-time data, revealing a trend of an increasing proportion of high-grade UTUC and minimally invasive surgery. Furthermore, urine B2-MG levels were significantly associated with DFS and MFS in this real-time evidence-based prospective registry, raising the evidence power.

This study included a well-designed single-center prospective cohort with all the procedures performed under the same protocol from the time of the first visit to diagnosis, treatment, and follow-up after treatment. However, this study had several limitations. First, although this study was performed using a prospective database, we had a relatively small cohort with insufficient time to analyze overall survival due to a lack of death events. Specifically, the number of cancer-specific deaths was 5 (1.7%), while deaths from other causes was 6 (2.0%). This is because patients have been enrolled in a prospective registry since 2016. Long-term follow-up of these patients may reveal a prognostic effect on overall survival in the future. Second, the pre-operative measurement of B2-MG revealed a significant association with disease recurrence and metastasis; nevertheless, this remains a description of the association. Time sequential measurement and co-measurement of other tubular injury markers, and measurement of serum levels of B2-MG, will provide more convincing results. In addition, randomized controlled trials should be conducted to support the clinical evidence of urinary B2-MG as a prognostic biomarker. Third, only 19.6% of patients have undergone regional lymph node dissection which could have led to inaccurate pathologic staging and could have affected oncologic outcome. Updated report suggest that lymph node dissection during nephroureterectomy provides prognostic data, and lymphadenectomy should be considered in high grade and large tumors (33). Fourth, as robotic surgery era has emerged, there is also updated issue of single stage robotic nephroureterectomy without re-docking (34), and its outcome. However, in our institution, information was limited and could not perform further analysis. Finally, although we considered the overall renal function of each patient by serum creatinine level and eGFR, we did not independently measure urine B2-MG for each side of the ureter or assess contralateral kidney function through the available methodology.

In conclusion, in patients with UTUC, increased urine B2-MG may represent the presence of advanced disease with a high tumor burden or aggressive tumor growth, which was ultimately associated with short DFS and MFS. This biomarker may aid in performing pre-operative risk stratification and assessing the individual prognosis of patients with UTUC.

## Data availability statement

The original contributions presented in the study are included in the article/supplementary material. Further inquiries can be directed to the corresponding author.

## Ethics statement

The studies involving human participants were reviewed and approved by The Institutional Review Board (IRB) of the Seoul National University Hospital. Written informed consent for participation was not required for this study in accordance with the national legislation and the institutional requirements.

## Author contributions

JK had full access to all the study data and takes responsibility for the integrity of the data and accuracy of the data analysis. Study concept and design: JK. Acquisition, analysis, or interpretation of data: JH and JK. Drafting of the manuscript: JH and JK. Statistical analysis: JH, SJ, SK. Administrative, technical, and material support: JH, CJ, CK, and JK. Study supervision: HY, CJ, CK, and JK.

## Conflict of interest

The authors declare that the research was conducted in the absence of any commercial or financial relationships that could be construed as a potential conflict of interest.

## Publisher’s note

All claims expressed in this article are solely those of the authors and do not necessarily represent those of their affiliated organizations, or those of the publisher, the editors and the reviewers. Any product that may be evaluated in this article, or claim that may be made by its manufacturer, is not guaranteed or endorsed by the publisher.
